# The Place of Research in Education

**Published:** 1896-01-11

**Authors:** 


### The Place of Research in Education.

"Science Progress" for January gives the place of honour in its pages to a paper on this subject by Professor H. E. Armstrong. By "research" Professor Armstrong means the acquiring of a theoretical and practical knowledge of a subject by personal work and experience. For research, as thus generally defined, he claims a place in British education; a place, that is to say, not only in our schools of all ranks and classes, but in every place where boys and girls, men and women, learn and work. But is not this kind of research everywhere carried on in schools, workshops, farms, dairies, medical consulting rooms, and throughout every department of English practical life? No, it is not, Professor Armstrong assures us. On the other hand, he affirms that in American schools, workshops, farms, and medical consulting rooms, and still more in all those of Germany, this kind of thorough research is carried out from the first to the last day of educational life and practical work. Because America and Germany practise this kind of research universally in their education they are getting far ahead of other nations in scientific competency and material wealth; and because Britain does not practise it, we are told, she is being beaten in one field of competition after another, until it appears probable that from a first-rate she will fall to a second-rate position in all the practical competitions of life.

Such, in briefest outline, is the substance of Professor Armstrong's thesis on the question of "The Place of Research in Education." What is to be made of it? On behalf of many of our fellow countrymen we plead guilty to the charge. We fear, however, that a plea of "guilty" will not satisfy Professor Armstrong. He insists that we are not only guilty but "impenitent." We are, he says, so "cock-sure," like the great Lord Macaulay, so certain of our own indisputable superiority to the foreigner, that nothing but a "sound whipping" in every trade and manufacturing competition we enter upon will be of the least service to us. That "sound whipping," he maintains, we have already received in several scientific and commercial fields; and from it he hopes for better results.

In truth we have no choice in this matter. We must be scientific: and we must not allow ourselves to think of science as something apart from practical life. It is one of our faults in this country that we have so thought of it; and for this many of our scientific men have themselves been to blame. They have insisted upon "science for the sake of science," and have scorned the non-specialist as a "mere amateur," even when he has been most willing to become an admiring and a humble disciple. All this must come to an end. If it be granted that our medicine, our commerce, and our manufactures must become scientific or perish, then it follows that a way must be found whereby theory and practice may meet together, science and education may kiss each other.

There is yet another aspect of the case. Science, or rather scientific teaching, is the most delightful part of education. There are even those who affirm that a scientific training is the best of all trainings for mental discipline. That we are not prepared fully to endorse; but that it is a training of a value which can hardly be over-estimated we most decidedly affirm. But this statement must be qualified, as Professor Armstrong qualifies it. He tells us that much of the science teaching in schools, most of it perhaps, is a mere pretence and waste of time. It is "cram" in its worst and most "soul-deadening" form. What he insists upon is that every schoolboy and schoolgirl, every apprentice in a workshop, every employer, foreman, and common workman shall use the principles and methods of science in daily life. What are those principles and methods? They are what we have already declared them to be, the principles and methods whereby every learner and worker finds out everything which can be known, both theoretically and practically, of the subject he is working at, by his own personal work and experience. In other words, Professor Armstrong denounces mere routine and "rule of thumb," and demands that every worker shall work with brains as well as hands, and with hands as well as brains.

